# Major inconsistencies of inferred population genetic structure estimated in a large set of domestic horse breeds using microsatellites

**DOI:** 10.1002/ece3.6195

**Published:** 2020-04-12

**Authors:** Stephan Michael Funk, Sonya Guedaoura, Rytis Juras, Absul Raziq, Faouzi Landolsi, Cristina Luís, Amparo Martínez Martínez, Abubakar Musa Mayaki, Fernando Mujica, Maria do Mar Oom, Lahoussine Ouragh, Yves‐Marie Stranger, Jose Luis Vega‐Pla, Ernest Gus Cothran

**Affiliations:** ^1^ Centro de Excelencia de Modelación y Computación Científica Universidad de La Frontera Temuco Chile; ^2^ Nature Heritage St. Lawrence UK; ^3^ Faculté des Sciences de la Nature et de la Vie Université d'El‐Tarf El‐Tarf Algeria; ^4^ Faculté de Pharmacie Université Laval Québec City QC Canada; ^5^ College of Veterinary Medicine and Biomedical Science Texas A&M University College Station TX USA; ^6^ Society of Veterinary, Environment and Agriculture Scientists (SAVES) Quetta Pakistan; ^7^ Ecole Nationale de Médecine Vétérinaire Sidi Thabet Tunisie; ^8^ Centro Interuniversitário de História das Ciências e da Tecnologia (CIUHCT) Faculdade de Ciências Universidade de Lisboa Lisboa Portugal; ^9^ Departamento de Genética Universidad de Córdoba Córdoba Spain; ^10^ Department of Veterinary Medicine Usmanu Danfodiyo University Sokoto Nigeria; ^11^ Instituto de Producción Animal Universidad Austral de Chile Valdivia Chile; ^12^ CE3C – Centre for Ecology, Evolution and Environmental Changes Faculdade de Ciências Universidade de Lisboa Lisboa Portugal; ^13^ Institut Agronomique et Vétérinaire Hassan II Rabat Morocco; ^14^ Marmande France; ^15^ Laboratorio de Investigación Aplicada Crıa Caballar de las Fuerzas Armadas Cordoba Spain

**Keywords:** domestic horse, population genetic structure, Przewalski horse, STRUCTURE analysis

## Abstract

STRUCTURE remains the most applied software aimed at recovering the true, but unknown, population structure from microsatellite or other genetic markers. About 30% of structure‐based studies could not be reproduced (*Molecular Ecology*, 21, 2012, 4925). Here we use a large set of data from 2,323 horses from 93 domestic breeds plus the Przewalski horse, typed at 15 microsatellites, to evaluate how program settings impact the estimation of the optimal number of population clusters *K*_opt_ that best describe the observed data. Domestic horses are suited as a test case as there is extensive background knowledge on the history of many breeds and extensive phylogenetic analyses. Different methods based on different genetic assumptions and statistical procedures (dapc, flock, PCoA, and structure with different run scenarios) all revealed general, broad‐scale breed relationships that largely reflect known breed histories but diverged how they characterized small‐scale patterns. structure failed to consistently identify *K*_opt_ using the most widespread approach, the Δ*K* method, despite very large numbers of MCMC iterations (3,000,000) and replicates (100). The interpretation of breed structure over increasing numbers of *K*, without assuming a *K*_opt_, was consistent with known breed histories. The over‐reliance on *K*_opt_ should be replaced by a qualitative description of clustering over increasing *K*, which is scientifically more honest and has the advantage of being much faster and less computer intensive as lower numbers of MCMC iterations and repetitions suffice for stable results. Very large data sets are highly challenging for cluster analyses, especially when populations with complex genetic histories are investigated.

## Graphical abstract

Very large data sets are highly challenging for cluster analyses, especially when populations with complex genetic histories are investigated. Here we use a large set of data from 2,323 horses from 93 domestic breeds plus the Przewalski horse, typed at 15 microsatellites, to evaluate how the settings of the program STRUCTURE, the gold standard for this type of analyses, impact the estimation of the optimal number of population clusters Kopt that best describe the observed data.

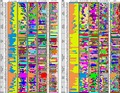

## 1 INTRODUCTION

Molecular ecology and conservation biology heavily rely on the identification of population structure and genetic admixture between individuals and populations. Model‐based inference of these parameters has become the central methodological approach especially for microsatellite markers (Pritchard, Stephens, & Donnelly, 2000). Bayesian statistics utilizing Markov Chain Monte Carlo, MCMC, simulations has been implemented in various computer programs such as baps (Corander, Marttinen, Sirén, & Tang, 2008) and structure (Pritchard et al., 2000). The latter is the by far most applied software for microsatellites (188 and 17,997 citations for baps and structure, respectively, in web of science by March 2019), acts as a benchmark, and remains popular even after the advent of alternative markers such as SNPs (1,188 structure citations for 2018 compared with peak citations of 1,433 for 2014). structure performs relatively well under various population models (Gilbert et al., 2012; Puechmaille, 2016; Putman & Carbone, 2014), but the reanalysis of structure‐based studies failed in ~30% of cases to reproduce results (Gilbert et al., 2012). Underpinning factors include the inherent stochastic nature of the model‐fitting procedure, nonconvergence of parameter estimates due to inappropriate number of MCMC iterations, inappropriate number of replicate runs *R*, weak population structure, too few informative microsatellite loci, and the estimation procedure per se (Gilbert et al., 2012; Putman & Carbone, 2014). The reanalysis used 34 studies with relatively small data sets (median = 254 individuals) including only one case with > 1,000 individuals. Three‐quarter of studies used less than the recommended *R* = 20 (Evanno, Regnaut, & Goudet, 2005; Gilbert et al., 2012). The review concluded that, in general, additional replicates are highly important to achieve higher precision of the parameter estimates (Gilbert et al., 2012). Replicates are essential to achieve higher precision of the parameter estimates including their variance, which are the basis for the ad hoc statistic Δ*K*, the dominant statistic to identify the optimal number of clusters that best explain the data (Evanno et al., 2005). In conjunction with the observation that structure performs best with a small number of discrete populations (Pritchard et al., 2000), the nonrepeatability might point to as yet not evaluated performance issues of structure and Δ*K* specific for large data sets.

Microsatellite data sets with large numbers of samples and animal populations have become increasingly available, especially in relatively easy to collect organisms such as near‐sedentary wild animals, for example, more than 2,500 colonies of the Broadcast spawning coral *Acropora tenuis* (Lukoschek, Riginos, & van Oppen, 2016), and domestic animals, for example, 1,514 dogs from 61 breeds (Leroy, Verrier, Meriaux, & Rognon, 2009), 1,826 sheep from 49 breeds (Leroy et al., 2015), 1,924 cattle from 40 breeds (Martin‐Burriel et al., 2011), 3,333 cattle from 81 breeds (Martínez et al., 2012), and 1,547 and 2,385 horses from 25 and 50 breeds, respectively (Cortés et al., 2017; van de Goor, van Haeringen, & Lenstra, 2011). While one study did not specify the number of repeats (Cortés et al., 2017), only low numbers of replicates ranging from *R* = 4 to *R* = 10 were employed except of *R = *50 by Leroy et al. (2009, 2015). Moreover, the choices of *R* and MCMC iterations are rarely justified in the literature. structure works best with relatively small numbers of demes or populations (Pritchard et al., 2000) but the performance with large data sets remains unevaluated. It is noteworthy that the largest study included in Gilbert et al. (2012) with 1,361 Anacapa deer mice (Ozer, Gellerman, & Ashley, 2011b) was nonrepeatable despite employing the recommended number of replicates. An evaluation of the performance of structure and ΔK for large data sets is thus important and timely.

Domestic horses (*Equus caballus*) lend themselves as an empiric test case to evaluate these questions not only because of our large sample size but also detailed information on natural history and breeding management of modern breeds is available. Horses were relatively late domesticated at ≈5.5 KYA, but then rapidly gained wide distribution due to their versatility (Levine, 2005; Librado et al., 2016; Warmuth et al., 2012). Widespread gene flow during and after domestication recurred from ancestral lineages including the Przewalski horse (*Equus przewalskii)*, the wild horse *Equus ferus,* and an extinct wild, taxonomically undescribed horse population into domestics and vice versa (Der Sarkissian et al., 2015; Schubert et al., 2014; Warmuth et al., 2012). A strong sex bias to the overall gene pool not only characterizes domestication but also modern breed management where it is exaggerated by the use of a small, selected number of stallions (Wallner et al., 2017). The formation of systematic modern horse breeding is marked by the establishment of the earliest studbook, that of the Lipizzaner in 1580 and continues till today with newly emerging formal breeds (Galov et al., 2013). The large variety of breed management is exemplified by the co‐existence of closed breeds without admixture from outside breeds, open breeds, landraces, and feral populations with little artificial selection but sometimes strong natural selection such as the adaptation to high altitudes (Hendrickson, 2013), and breeds with strong artificial selection for desired traits. Two large microsatellite studies investigated 67 and 41 breeds, respectively, and constructed distance‐based phylogenies using a variety of phylogenetic methods (Conant, Juras, & Cothran, 2012; Pires et al., 2016). In general, these approaches revealed patterns largely reflecting known breed histories, but statistical support was consistently low (Cothran & Luís, 2005). Whether these low bootstrap values are a consequence of complex breed history and admixture or caused by the phylogenetic methods per se (Pardi & Scornavacca, 2015; Pritchard et al., 2000) remains unknown.

Using large‐scale screening of domestic horse breeds alongside the Przewalski horse, we aimed to evaluate the robustness of structure and ΔK with emphasis on the effects of the numbers of MCMC iterations and replicates. Results are compared with alternative clustering methods based on different model assumptions. structure assumes linkage equilibrium, LE, and Hardy–Weinberg equilibrium, HWE (Pritchard et al., 2000). In contrast, the multilocus maximum likelihood approach (Paetkau, Calvert, Stirling, & Strobeck, 1995) as implemented in FLOCK (Pierre Duchesne & Turgeon, 2012) iteratively allocates genotypes into genetic clusters without assuming HWE and LE. Discriminant analysis of principal components, DAPC, as implemented in the software adegenet (Jombart, 2008; Jombart, Devillard, & Balloux, 2010) is a model‐free multivariate method.

## 2 MATERIALS AND METHODS

### 2.1 Samples and genotyping

A total of 4,392 individuals from 94 horse populations (Table S1) were collected during long‐term studies on horse genetics (Cothran, Canelon, Luis, Conant, & Juras, 2011; Cothran & Luís, 2005; Khanshour, Conant, Juras, & Cothran, 2013; Khanshour, Juras, Blackburn, & Cothran, 2015; Khanshour, Juras, & Cothran, 2013; Luís, Cothran, & Oom, 2007; Pires et al., 2014). The sample includes the Przewalski horse and 89 internationally recognized breeds of which Arabian horse, Barb horse, and Spanish Pure Breed stem from five, three, and two different management schemes and studbooks, respectively. North American bred Spanish Pure Bred is commonly called Andalusian. Jointly, they represent 93 domestic breeds sensu lato plus the Przewalski, henceforth all denoted as “breed” for simplicity. The geographic origin of breeds was focussed on South and Central America (*n* = 13), the Iberian Peninsula (*n* = 14), the British Isles (*n* = 16), and Central and Eastern Europe (*n* = 18) with additional, representative breeds from North America (*n* = 5), Southeastern Europe (*n* = 7), Africa (*n* = 10), and Asia (*n* = 10). Horse breeds include highly divergent breeding strategies and histories including cold‐blooded, Celtic and Iberian horses in Europe, and Arab and non‐Arab horses in Africa (Table S1). Breeds were labeled in the figures according their geographic origin rather than their breeding history as a neutral approach to visual interpretation of the graphs. Except for four breeds with less than 25 individuals each, we reduced sample sizes to 25 individuals per breed in order to account for the impact of uneven sampling that tends to bias the evaluation of population genetic structure (Puechmaille, 2016). In horses, sample sizes of 25 per breed have been demonstrated suitable for evaluating population genetic structure (Cothran & Luís, 2005). Individuals were excluded in order to remove horses from the same group or farm, which may be distantly related, although great care was taken not to sample relatives. In total, 2,323 horses were included in the analysis (Table S1).

We used 15 autosomal microsatellite markers, distributed on 14 chromosomes, from marker panels that are recommended for diversity studies by ISAG‐FAO and the International Society for Animal Genetics (Cothran & Luís, 2005; FAO, 2011). Details on genotyping can be found in Juras, Cothran, and Klimas (2003) and Khanshour, Conant, et al. (2013).

### 2.2 Information content of the genetic data

The combination of sample sizes, numbers of microsatellite loci, numbers and frequencies of observed alleles, and the degree of differentiation between individuals and populations, typically quantified by *F*_IS_ and *F*_ST_, establishes the information content and impacts the statistical power to detect genetic structure and differentiation (Ryman et al., 2006). We evaluated it with a simulation approach implemented in powsim with observed allele frequencies, 1,000 replicates, 10,000 burn‐ins, and 100 batches with 10,000 iterations (Ryman, 2011; Ryman & Palm, 2006). Because the published version is limited to 30 populations, we also used a version adjusted to our data set permitting 90 breeds (Ryman, M. Stephan, S. M. F. Funk, personal communication). To generate expected *F_ST_* values, a generation time of 5 years since domestication ≈5,500 KYA was used. Considering that generation time may vary largely and that five to 12 years has been cited (Lorenzen et al., 2011; Sokolov & Orlov, 1986), our choice of five years is conservative and allows drift over 1,100 generations. We selected a range for effective population size, *N*_e_, that reflects the evolution of modern breeds by varying *N*_e_ between 10 and 1,000, resulting in expected *F*_ST_ values, estimated by POWSIM, between 0.001 and 0.87. This range of simulated *F*_ST_ values included all observed values of investigated horses. Power was estimated using Fisher's exact and chi‐square tests at 95% confidence interval (Ryman & Palm, 2006). Power at *t* = 0 (*F_ST_* = 0) represents the type I error, the frequency that genetic homogeneity is rejected while it is true. The information content was also evaluated using the clustering program flock (Duchesne, Méthot, & Turgeon, 2013; Duchesne & Turgeon, 2012) which designates an “undecided stopping condition” when an optimal number of population clusters explaining the data cannot be identified. Two underpinning scenarios are possible: lack of “true” genetic structure or insufficient genetic information content. Discriminating these scenarios is part of the interpretation of the FLOCK results described further below in the context of population clustering.

### 2.3 Hardy–Weinberg equilibrium and F‐statistics and PCoA analysis

genepop 4.5.1 (Rousset, 2008) was used for exact HWE tests for all locus/population combinations employing Markov Chain permutations (Guo & Thompson, 1992) with 10,000 de‐memorizations and 400 batches with 10,000 iterations each. Fisher's combined probability test was used to calculate *P* across loci and across populations, respectively. For the global analysis, *P* was calculated with and without adjusting for multiple testing by sequential Bonferroni correction (Holm, 1979). *F*_IS_ was calculated according Weir and Cockerham (1984). Genetic differentiation was evaluated by pairwise weighted mean *F*_ST_ distances between breeds (Cockerham & Weir, 1993) and the log‐likelihood ratio, G, test in fstat 2.9.3 (Goudet, 1995) with 10,000 randomizations of genotypes without assuming random mating. Genic differentiation was evaluated by exact G and Fisher's combined probability tests in genepop 4.5.1 (Rousset, 2008). The *F*_ST_ matrix was summarized by principle coordinate analysis, PCoA, with covariance standardization in genalex 6.502 (Peakall & Smouse, 2012). Linkage equilibrium, LE, was not evaluated as it was not rejected for the same loci in several previous studies (e.g., Khanshour et al., 2015).

### 2.4 STRUCTURE analysis

structure 2.4.4 (Pritchard et al., 2000; Pritchard, Wen, & Falush, 2012) was applied allowing for correlated allele frequencies and admixture permitting mixed ancestry and accounting for recent between‐breed gene flow. The locprior model with breed assignment as prior information, which is more sensitive for detecting weak population structure than the nonlocprior model, was used alongside the latter as recommended to check for consistency (Hubisz, Falush, Stephens, & Pritchard, 2009). We denote the models as +LP and −LP henceforth. Gilbert et al. (2012) recommend at least MCMC 100,000 iterations and, ideally, larger runs lengths, but these are often limited by run‐time considerations. Three sets of MCMC iterations were run for each of the ±LP models: 150,000, 750,000, and 1,500,000 of which the first 50,000, 250,000, 500,000, and 1,000,000 iterations, respectively, served as burn‐ins. Because of computing time restrictions, the scenario with 3,000,000 MCMCs, including 1,000,000 burn‐ins, was restricted to the +LP model. A priori defined clusters, *K*, were considered from *K = *2 to *K* = 30 in steps of one and from *K = *35 to *K = *90 in steps of five. For each *K* and each MCMC/LP scenario, one of the replicates was randomly selected for visual inspection of the Dirichlet parameter *α*, which estimates the degree of admixture, and the log‐likelihoods for convergence (Gilbert et al., 2012; Pritchard, Wen, & Falush, 2010). We restricted ourselves to the visual inspection as STRUCTURE has not implemented any formal assessment of convergence nor have any respective statistics such as Gelman and Rubin's statistics (1992) been implemented and tested for STRUCTURE (e.g., Zachos et al., 2016). STRUCTURE reports the posterior probability of the data for a given *K* for each replicate, denoted as Pr(X|*K*) in Bayesian notion and abbreviated as *P(K)* henceforth. For all MCMC/±LP scenarios, *R = *40 replicates were used except for *R = *100 for the 1,500,000 MCMC/‐LP scenario. Computations used the sgi uv‐2000 intel(r) xeon(r) e5‐4640 @ 2.40ghz (sandybridge) computing platform with 96 nodes, 192 cores, and 1.5 teraflop.

The most likely number of clusters that best describe the given data was first estimated by the visual inspection of the means and standard deviations of *P(K*) over all investigated *K* according to Pritchard et al. (2000), Pritchard et al. (2010), henceforth denoted *K*_opt[Pritchard]_. Second, structure harvester v. 0.6 (Earl & von Holdt, 2012) was used to calculate the optimal *K* according the Evanno Δ*K* statistics, henceforth denoted *K*_opt[Evanno]_. We evaluated the convergence of the two estimates over *r* by first partitioning all *R* replicates into *B* subsequent blocks of 5 replicates each. *P(K*), *K*_opt[Evanno]_, and *K*_opt[Pritchard]_ were then calculated for both, each block separately and accumulated over all preceding blocks.

In order to evaluate the impact of *R* in smaller data sets on the optimal *K* selection, a subset of 295 individuals from five Arab breeds, Algerian, Moroccan and Tunisian Barb, Akhal Teke, Caspian, Turkoman and the Przewalski horse was analyzed with *R = *40. A similar selection of breeds from the same data base was used previously with *R = *10 and 120,000 MCMCs and 200,000 MCMCs, respectively (A. Khanshour, Conant, et al., 2013). Alongside, data on 1,361 Anacapa deer mice (Ozer, Gellerman, & Ashley, 2011a; Ozer, Gellerman, & Ashley, 2011b) were also re‐evaluated with the originally applied STRUCTURE parameters, but with *R = *100.

Whether all *P(K)* values resulted in similar or multimodal solutions was evaluated on the clumpak 1.1 webserver (Kopelman, Mayzel, Jakobsson, Rosenberg, & Mayrose, 2015, http://clumpak.tau.ac.il). All MCMC scenarios were combined for each LP model. Settings utilized the largekgreedy algorithm (Jakobsson & Rosenberg, 2007) and dynamic threshold determination (Kopelman et al., 2015). We then re‐calculated ∆*K* and *K*_opt[Evanno]_ using only the dominant solutions, that is, the “major modes” (Kopelman et al., 2015), and excluded “minor modes,” rare different solutions when present. The visual presentation of the cluster's estimated membership coefficients for individuals and breeds utilized distruct 1.1 (Rosenberg, 2003) with replicates from the majority modes.

We identified “ghost clusters” which are spurious clusters that the MCMC search strategy produces. They occur when the a priori *K* is larger than the optimum with structure enforcing suboptimal, not empty clusters. Following Guillot, Mortier, and Estoup (2005), Puechmaille (2016) defines ghost clusters as those with mean membership coefficients <0.5 in any studied population and then subtracts the number of ghost clusters from the ∆*K* estimate.

### 2.5 FLOCK analysis

flock 3.1 (Duchesne et al., 2013; Duchesne & Turgeon, 2012) was applied using 20 re‐allocations for the multilocus maximum likelihood procedure with 50 runs for each *K* and a LLOD threshold score of 0. The optimal number of clusters, *K*_opt[FLOCK]_, was determined by ad hoc “stopping” rules (Pierre Duchesne & Turgeon, 2012). Log‐likelihood scores, LLOD(*K*), averaged over genotypes, and replicates are sorted according their absolute values over all runs. "Plateau lengths” are the counts of scores with the same values and are the core of the “stopping rules.” Hierarchical analysis was applied for each of the identified breed clusters until each cluster could not be further subdivided. The total number of undividable clusters identifies *K*_opt[FLOCK]_.

In cases where flock produced an “undecided stopping condition,” the underpinning cause was evaluated in three steps, which are not described in the original publication or the flock manual (Duchesne et al., 2013; Duchesne & Turgeon, 2012). These steps aim to distinguish between insufficiently variable microsatellite data and the level of genetic structure as possible causes. The latter case is produced when relatively many highly admixed individuals and/or breeds are present which flock cannot consistently assign to single clusters because of stochasticity (Duchesne M. Stephan, S. M. F. Funk, personal communication). First, the “Samples likelihood maps” in FLOCK's output, that is, the averaged likelihoods LLOD(*K*) in each breed, were visually inspected for clear outlier breeds showing values of a different magnitude than all other breeds. Because of differential effects of stochasticity, LLOD(*K*) values among nonoutliers may be deflated when outliers are present, leading to an “undecided stopping condition” (Duchesne, M. Stephan, S. M. F. Funk, personal communication). Outliers and nonoutliers were separated, and then, both sets were separately re‐analyzed. Second, when no outliers were identifiable for an “undecided stopping condition,” the “Sample allocation matrix” in FLOCK's output, that is, the allocation of samples to clusters, was evaluated. Breeds with less than 80% of horses allocated to a single cluster were removed from the hierarchical analysis as “admixed.” After reanalysis of the remaining breeds, two outcomes are possible: If an “undecided stopping condition” emerged again, then the genetic information content of the microsatellite markers is insufficient to reveal genetically differentiated clusters (Duchesne, M. Stephan, S. M. F. Funk, personal communication). If a stopping condition was reached, the previous failure to detect structure indicates that a relatively high number of admixed individuals and/or breeds in the given data set prevented the identification of clusters. Third, the “admixed” breeds identified in the previous step were added stepwise to the reanalysis in order to evaluate whether single breeds or a combination of breeds resulted in the “undecided stopping conditions.”

### 2.6 DAPC analysis

The DAPC analysis utilized the adegenet 2.0.1 module for the r environment (Jombart et al., 2010; Jombart et al., 2016). We used r 3.3.1 (R Foundation for Statistical Computing, 2016). The first step is to transform all genotypes into noncorrelated variables using a principle component analysis, PCA. For downstream analysis, all principle components, PCs, were retained. After PCA, discriminant analysis, DA, is applied. It partitions the variance within and between a priori defined clusters, *K*, such that the separation between clusters is maximized. We evaluated *K* for 2 to 94, the total number of breeds. To prevent overfitting by DAPC, 50 PCs, approximately one‐third of the total number of PCs identified by the PCA model was used (Jombart & Collins, 2016). The optimal *K,* denoted *K*_opt[DAPC]_ henceforth, was chosen by visually and statistically identifying sharp changes in the Bayesian information criterion, BIC, over increasing *K* (Jombart et al., 2016). BIC quantifies the fit of the DAPC model at each *K*. adegenet's option to statistically identify “sharp” changes by Ward's clustering method (Mojena, 2006) was used. DAPC results were visualized as scatter plots in adegenet. Individual posterior memberships for cluster assignments were averaged for breeds and plotted analogous to structure using distruct 1.1 (Rosenberg, 2003).

## 3 RESULTS

### 3.1 *HWE and F*_IS_

A total of 23 from 1,410 locus/breed combinations revealed *p* = 0 for the exact HWE tests whereby three breeds had more than one locus involved (Timor Pony, Gotland, and Friesian). Fifteen breeds exhibited Fisher's *p* < .0001 over all loci (Table S1). Excluding the latter, two loci produced *p* < .001 (HTG10 and HTG7), but both their Bonferroni corrected *p*' values were > 0.05. Mean ± *SD* *F*_IS_ was 0.03 ± 0.17 over all breeds with a breed *F*_IS_ range of 0.2 to −0.11. The only breed with a mean ± *SD* range that did not include zero was the Timor Pony, which also exhibited the largest number of loci with *p* < .0001 for the HWE tests.

### 3.2 Breed differentiation

The global breed differentiation was highly significant (*p* < .0001, log‐likelihood *G* test). Estimated pairwise *F*_ST_ values between breeds ranged from 0.04 (Tushuri Cxeni—Pindos Pony) to 0.45 (Friesian—Abaco Horse) with a mean *F*_ST_ ± *SD* of 0.115 ± 0.064 among the 93 domestic breeds and 0.212 ± 0.037 for Przewalski versus the domestics (Table S2). Three domestics showed mean *F*_ST_ > 0.2 versus all other domestics (Abaco, 0.310 ± 0.041; Friesian, 0.259 ± 0.043; Sorraia, 0.235 ± 0.0.045; all highly inbred breeds). The 4,370 pairwise comparisons between breeds for genic differentiation revealed Fisher's *p* < .000001 in almost all comparisons except for 12 pairs with .007 > *p*>.000001. Eleven of the latter involved Arab horses bred in Chile and four African (Ethiopian, Moroccan Barb, Nigerian, and Tunisian Barb), two Asian (Pakistani and Kurdish), and four European (Pindos Pony, Tushuri Cxeni, Hanoverian, and Wielkopolski). Within these breed groups, there is shared common ancestry, for example, Hanoverian and Wielkopolski are both warmblood which have a Thoroughbred cross in their background.

**Figure 1:**
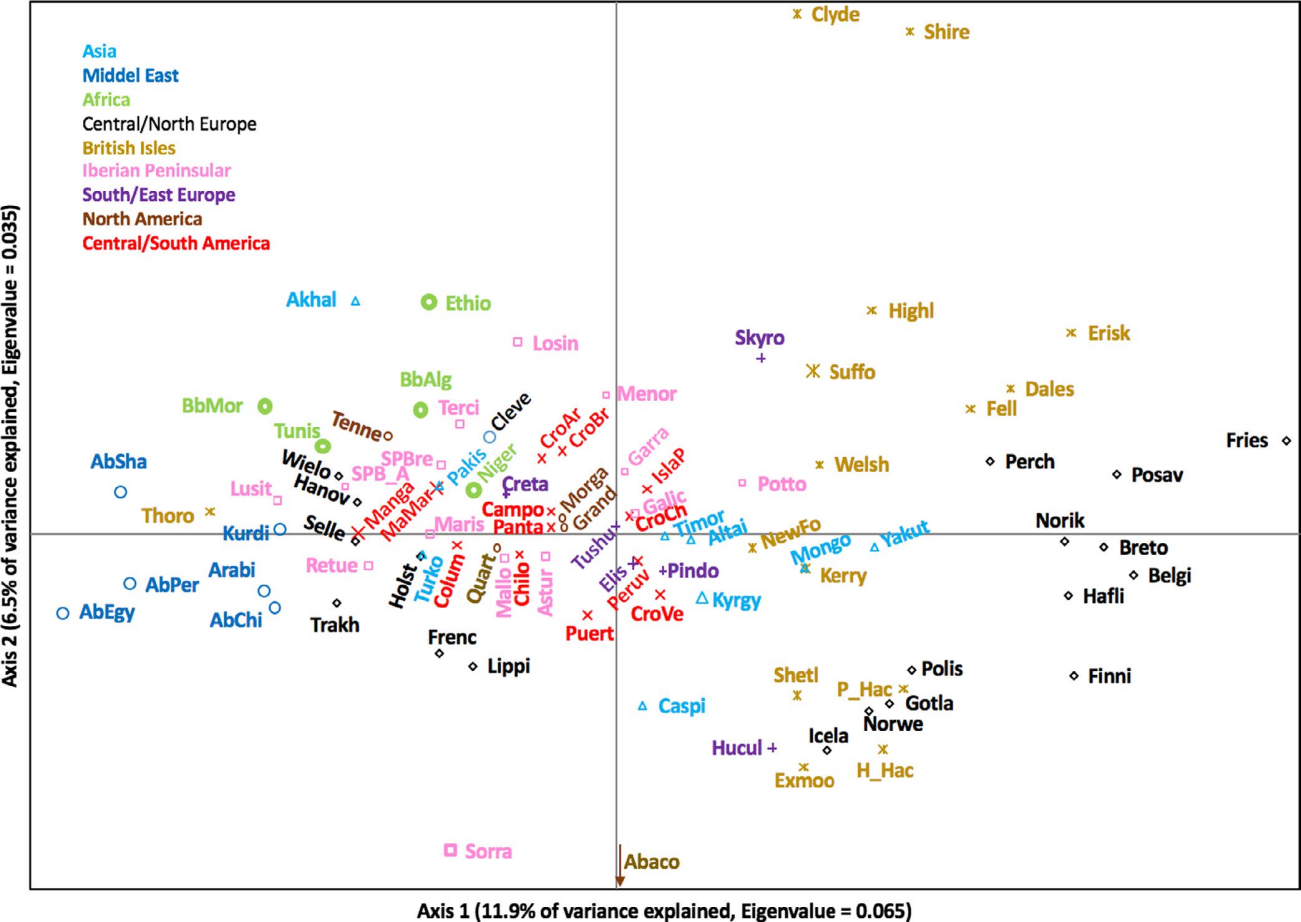
Scatter plot of the first three PCoA axes of genetic variation estimated by pairwise F_*ST*_ between breeds. Colour codes correspond to geographic origin and abbreviations to breeds as defined in Table S1, Supporting information

Figure 1 displays the PCoA matrix of pairwise *F*_ST_ values between breeds for the first two axes explained prearranged by geographic origin. The first two axes explained 18.3% of the total variation and the third axis added 5.4%. Visual inspection of the 1st‐versus‐2nd and 1st‐versus‐3rd component plots did not unequivocally separate clusters but there are clear centers of clustering according geographic origin and horse type. No clear overall geographic distribution of scores was seen. The scores on Axis 1 show fairly good correspondence to the breed groups that the breeds best fit rather than geography in most cases. The breeds furthest to the right are primarily the “cold‐blood” breeds composed of draft horses and ponies. The center part of Axis 1 is mainly composed by Iberian breeds or Iberian origin including those from the Americas while those on the left side are the Arabian breeds and also breeds with a strong Thoroughbred background such as the Wielkopolski, Hanoverian, Selle Français, and Trakehner. Separated from the others is also an ark formed by African and Asian horses. Axis two appears to best separate the Asian breeds with distribution of scores near the center. The Akhal Teke and Caspian are outliers of this group because of low diversity due to recent bottlenecks. The Abaco horse is the most pronounced outlier reflecting its breed history with a strong recent population bottleneck and a very low population size. The broad pattern seen in Figure 1 reflects well the relations among most of the breeds as known from breed histories.

### 3.3 *Statistical power and F*_ST_

POWSIM estimated that there was power of 100% for all test scenarios to reject genetic uniformity at *F*_ST_ ≥ 0.0025, which is lower than the smallest observed pairwise *F*_ST_ = 0.004 between breeds (Figure S1). The probability to falsely reject uniformity was low (*p* < .08) for all test scenarios, which is higher than 5% but normal for microsatellite markers (Ryman et al., 2006).

### 3.4 STRUCTURE analysis

A limiting factor for the choice of *R* and MCMCs was computing time, which was exceptionally large for the large data set. One replicate of 1,500,000 MCMCs for all *K* took ≈17.5 days for −LP and ≈49 days for +LP, respectively. Total computing time for all scenarios and *R* was ≈18.5 years, which was only achievable using up to 80 computing cores simultaneously.

**Figure 2:**
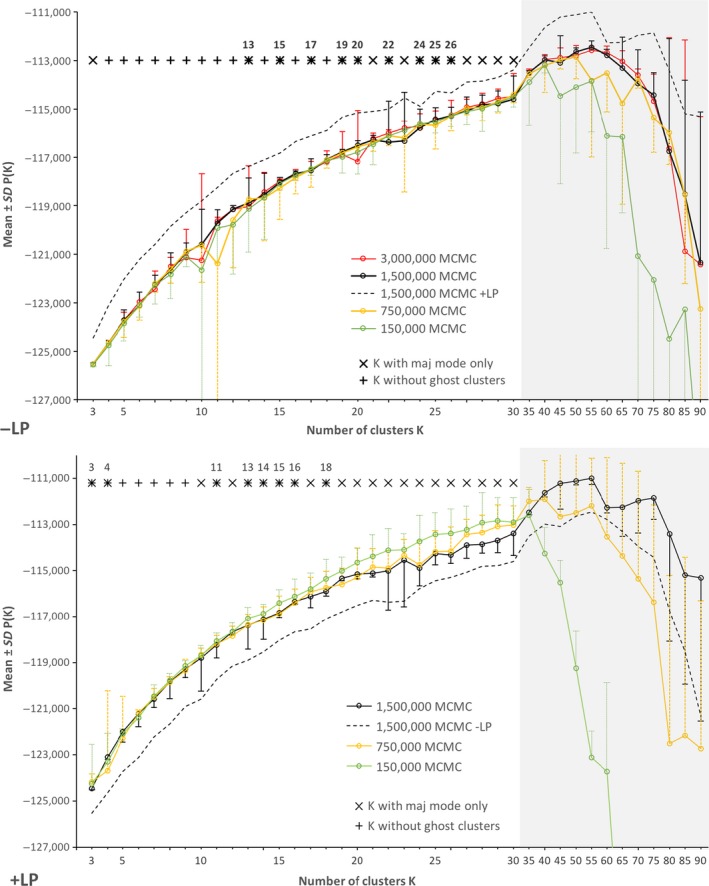
Log likelihood *P(K)* values from the Bayesian clustering approach for explored cluster numbers K for the models with locprior and without locprior. Clusters were explored stepwise from *K*=3 to *K*=30 and then in steps of 5 up to *K*=90 (indicated by the background in grey). Shown are the averaged ±SD *P(K)* values for 40 replicates at each MCMC scenario. At the top of each graph those *K* values, which only exhibited major modes and did not show ghost clusters, are indicated and the *K* number is shown where both conditions were fulfilled (see also Fig S2, Supporting information)

Mean *P(K) *values reached a plateau at around *K = *45 of each of the MCMC/LP scenarios, indicating an optimal cluster number of *K*_opt[Pritchard]_ ≈ 45 (Figure 2). The visual inspection of summary statistics of MCMC runs indicated convergence of the model parameters. For all MCMC/±LP scenarios, plots of mean ± *SD* were very similar with three exceptions. First, large variances occurred only for −LP at around *K* = 10 to *K* = 15, indicating that MCMC model failed relatively often to find similar solutions in this region of *K* values. Second, at *K* > 40, the short MCMC/‐LP scenario produced consistently lower (worse) *P(K)* values than the three scenarios with more MCMC iterations, whereas the 1,500,000 MCMC/+LP model consistently produced the highest (best) values. In other words, once the *P(K)* plateau is reached, the smallest MCMC regime performed worst. Third, the + LP scenario produced consistently higher *P(K)* values and therefore performed best compared to the –LP scenario. Pearson's pairwise correlations of mean *P(K)* up to *K* = 30 were all very high (*r* > .99) but low for variances (mean *r* ± *SD *= 0.29 ± 0.27 for six –LP pairs and mean *r* = .30, .16 and −.04 for the three +LP pairs, respectively).

**Figure 3:**
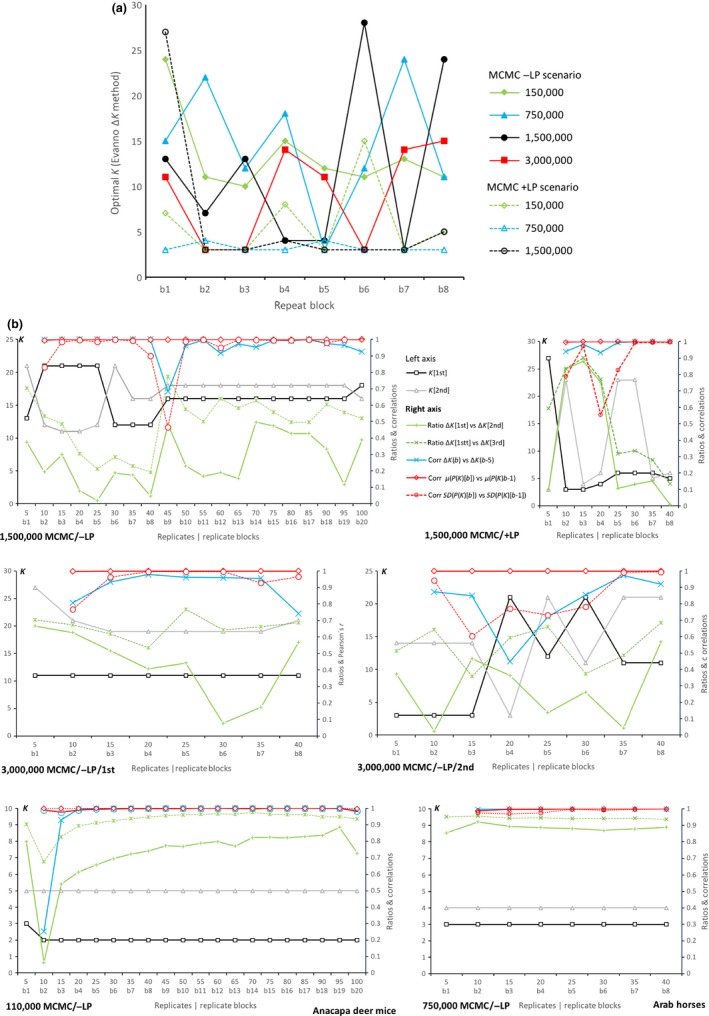
Estimation of the optimal number of clusters *K_opt[Evanno]_* using the Δ*K* method (Evanno et al., 2005) for different numbers of replicates. The log likelihoods *P(K)* and the Δ*K* values were calculated for *K*=2 to *K*=30. The number of replicates was subdivided into blocks consisting 5 replicates each. Panel A shows *K* for each of eight blocks b for different MCMC /±LP scenarios which used 40 replicates each. Panel B shows parameters of the Δ*K* method over accumulated blocks for up to 20 blocks (b1 with 5 replicates up to b20 with 100 replicates). Plotted parameters are the highest, the second and third highest Δ*K* peaks (*K*[1^st^ ] = K_opt[Evanno]_, K[2^nd^] and K[3^rd^]), the ratios between the heights of these peaks, the correlations for the mean ±SD *P(K)* values over *K* (μ *P(K)* and SD *P(K)*), and the Δ*K*values between each current b versus the previous *b*‐1. For the whole horse data set, the graphs for the 1,500,000 MCMC /‐LP scenario, the 1,500,000 MCMC /‐LP scenario, and for two iterations of the 3,5000,000 MCMC /‐LP scenario are shown. For Arab horses, the results for the 750,000 MCMC /‐LP scenario and for the Anapaca deer mouse dataset the results for the 110,000 MCMC / ‐LP set (i.e. the STRUCTURE settings used in the original analysis by Ozer et al., 2011a) are shown. In each case, 40 replicates were conducted except for the the 1,500,000 MCMC /‐LP scenario with 100 replicates

The Evanno method did not converge to single maximum Δ*K* value for any of the investigated scenarios. The consistency of the mean and standard deviations of *P(K)*_MCMC, ±LP_ between replicate blocks, *b*, is shown in Figure 3a. Mean *P(K)*_b, MCMC, LP_ values are highly correlated between replicate blocks *b* for all MCMC/LP scenarios (Pearson's *r* = .99 across all 49 pairwise comparisons, ±*SD* = 0.02). Thus, a single block of 5 replicates is sufficient to consistently estimate mean *P(K)*. In contrast, standard deviations varied largely and the mean ± *SD* Pearson's correlation coefficient *r* across all 49 pairwise comparisons was *r = *.14 ± .24, demonstrating large impacts of stochasticity on mean *P(K)* over *R* (Figure S2). For all scenarios, *K*_opt[Evanno]_ varied over accumulated blocks *b*.

**Figure 4:**
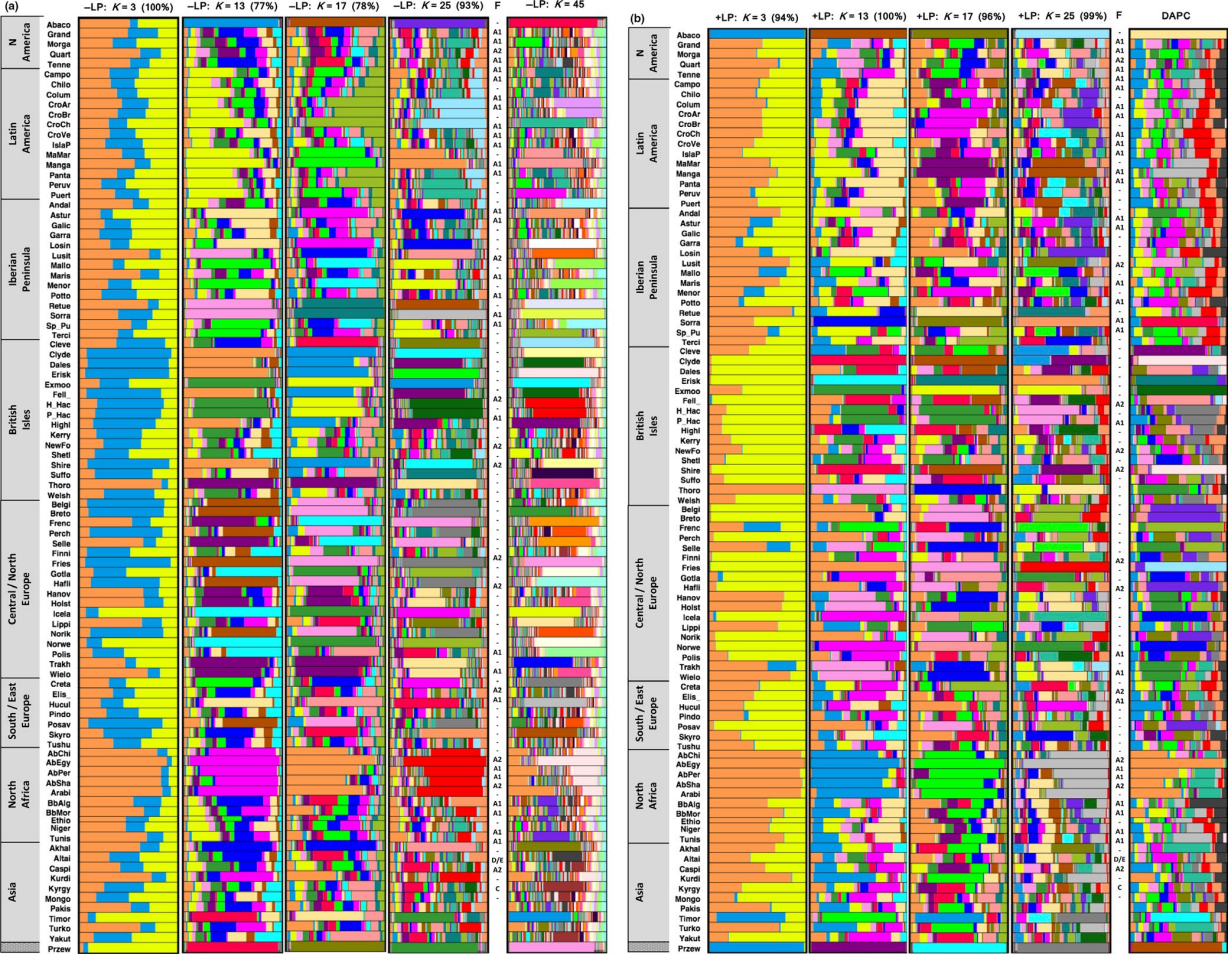
Membership coefficients for horse breeds from STRUCTURE at various *K* and ±LP models, posterior probabilities for DAPC cluster assignments at the optimal *K_opt[DAPC]_* =27, and summary of FLOCK breed cluster assignments. Panels for STRUCTURE are specified by the ±LP models, visualize membership coefficients within each genetic cluster as indicated by different colours. The membership coefficients shown were averaged over all horses within each breed. With the exception for _K_=45, the membership coefficients for each horse are averages for all replicates found in the major modes as estimated by CLUMPAK. CLUMPAK analysed 160 replicates stemming from 40 replicates from each MCMC/–LP scenario and 120 replicates stemming from 40 replicates from each MCMC/+LP scenario. The percentages of replicates within the major clusters are presented in brackets. All these presented panels correspond to *K* without ghost clusters and with only majority modes (Fig. 2). The exception K=45 refers to the estimated optimum, *K_opt[Pritchard]_* =45, which was inferred by the visual Pritchard method and includes ghost clusters. Major mode could not be calculated as the data volume for *K*=45 could not be processed by CLUMPAK. Therefore, the membership coefficients are visualized for the replicate with the best P(*K*=45) score. The column for FLOCK (F) refers to the breed assignment at the first hierarchical level and denotes the breed groups as presented in Fig. 5. For visual purposes, column F is shown jointly with the STRUCTURE –LP and +LP panels, respectively. The panel for DAPC cluster assignments are based on refer to the posterior probabilities for the optimal *K_opt[DAPC]_* =27 (Fig, 6), averaged for each breed

The 3,000,000 MCMC/‐LP scenario appeared an exception with an unchanging *K*_opt[Evanno]_ = 11 (Figure 3b) but varied greatly when the sequence of *b* was randomly iterated. In other words, even with double the number of the currently recommended *R* = 20, the Evanno method did not result in converged *K*_opt[Evanno]_ estimates. Because there is no difference of membership in major modes of the 1,500,000‐MCMC/–LP and the 3,000,000‐MCMC/–LP scenarios (see below), we used 500,000 MCMCs to evaluate convergence for *R* = 100 (Figure 4). From *b* = 9 (*R* = 45) up to *b* = 19 (*R* = 95), *K*_opt[Evanno]_ remained stable, but changed at *b* = 20 again. The ratios between maximum and second highest Δ*K* peaks varied largely even when *K*_opt[Evanno]_ remained unchanged. This ratio is an indicator for the impact of the variance of *P(K)* on Δ*K* estimates and is more sensitive for the variance than *K*_opt[Evanno]_.

In contrast to joint analysis of all breeds, *K*_opt[Evanno]_ for the subset of only 11 breeds converged by the second block (Figure 3b). The *K*_opt[Evanno]_ for re‐analyzed Anacapa deer mice (Ozer et al., 2011a) converged by the third repeat block at *K*_opt[Evanno]_ = 2. This partition corresponds with the geographic location of the 11 population samples, with one sample for the mainland and 10 samples of three island populations at and after release. Contrasting the large horse data set, the ratios between the highest versus the second highest and third highest ∆*K* peaks, respectively, and the correlations between subsequent repletion blocks of both cases showed very little variation after their respective stable *K*_opt[Evanno]_ values were reached.

clumpak identified major and minor modes without a bias toward a particular MCMC/±LP scenario (Figure S3). Only the +LP model at *K = *2 clustered the 150,000 MCMC results separate from the two other scenarios. Numbers of replicates within major modes correlated strongly between MCMCs for both the −LP model (Pearson's *r* = .94, *r* = .97 and *r* = .97 for 3,000,000 MCMCs versus 150,000 MCMCs, 750,000 MCMCs and 1,500,000 MCMCs, respectively) and the –LP model (*r* = .97 and .99 for 1,500,000 MCMCs versus 150,000 MCMCs and 750,000 MCMCs, respectively; excluding *K* = 2)*.* Both LP models produced exclusively major modes when *K* was relatively large (*K* > 17 and *K* > 9 for −LP and + LP, respectively) but added minor modes at lower *K*. The re‐application of the Evanno method using major modes only did not result in consistent *K*_opt[Evanno]_ estimates (Figure S4).

While maximum cluster membership coefficients remained high with increasing *K*, median memberships dropped to approx. 75% and 55% for the −LP and +LP models, respectively (Figure S3). The stronger drop for the +LP model is reflected in the rise of the number of ghost clusters starting at lower *K* than that for −LP. For the −LP and +LP models, all *K* > 26 and *K* > 18, respectively, exhibited ghost clusters while lower *K* only rarely had them. A peak of ghost clusters was found at *K* = 10 for + LP, which parallels the large variances of mean *P(K)* at the same *K* (Figure 2). Because occurrences of minor modes and ghost clusters were reversely distributed over *K*, only a relatively small number of *K* was without both (Figure 2). Nine such clusters without minor modes and ghosts ranged between *K* = 13 and *K* = 26 for −LP. For + LP, the range occurred at lower *K*, namely eight clusters between *K* = 3 and *K* = 18.

Cluster membership coefficients at various *K* values are visualized in Figure 4 for the two LP models prearranged by geographic origin. At *K* = 3, the resolution is very low for both models although the +LP model indicates some distinctive and homogeneous patterns including “Cold‐blood” breeds, for example, Eriskay Pony and Belgium Draft, and breeds that are associated with the Arab horse, for example, Egyptian Arab and Thoroughbred. Only +LP separates the Przewalski horse and the Abaco, the most inbred domestic horse in our sample, from all others. This separation becomes evident by *K* = 13 for −LP. With increasing *K*, several patterns emerge:

First, breeds separate into two types: those which remain relatively homogenous and those which become more and more fragmented into an increasing number of clusters. These highly homogeneous breeds are Abaco, some Iberian breeds (Retuertas, Sorraia), some “Cold‐blooded” breeds from the British Isles (Clydesdale, Exmoor, Shire), some Central and North European breeds (Belgian, Breton, Haflinger, French Trotter, Friesian, Icelandic, Norwegian, Trakehner, Wielkopolski), the Arabian breeds, some Asian breeds (Kurdish, Timor), and the Przewalski.

Second, regional trends and genetic similarities between breeds with shared breeding history emerge. By *K = *7, geographic clustering has emerged in both LP models whereby the American and Iberian breeds tend to cluster jointly. By *K* = 13, European breeds are highly differentiated on a local/ regional scale including two groups of local breeds from the British Isles, three groups in central‐North Europe, two groups in Africa and Asia, and several highly admixed breeds in all areas especially in Southeast Europe. This differentiation is stronger for −LP than + LP. However, a substantial number of breeds did not show any clear‐cut trend of separation, thus reflecting substantial admixture.

Third, differences between the two LP models emerge, for example, the American and Iberian breeds roughly split into North American, South American, and Iberians by *K* = 13 for −LP while no such structuring becomes evident for +LP. Iberian and American breeds are more fragmented within breeds for +LP than for −LP. Pronounced differences include the Clydesdale, Dales, and Eriskay group, which is tightly linked up to *K* ≈ 17 for −LP but disassociated by *K* = 13 for +LP, respectively. The Dales and Exmoor pony and the Haflinger are among those breeds which remain genetically homogenous up to *K* = 45 at −LP (excluding many ghost clusters) but clearly fragmented at the same *K* for +LP.

Fourth, adding clusters with smaller membership coefficients does not follow a linear pattern over increasing *K* for some breeds. For example, Przewalski and Timor Pony cluster separated for −LP at *K* = 7, joint at *K* = 13, separated at *K* = 17, and joint again at *K* = 27. Cleveland Bay fragments up to around *K* ≈ 13 and homogenizes at *K* ≈ 17 and higher for −LP, and Exmoor fragments up to *K* ≈ 10 and homogenizes at *K* ≈ 13 and higher for both locprior scenarios.

Lastly, the emergence of ghost clusters as shown by the quantitative analysis (Figure S3) becomes visually evident by *K* ≈ 27 and is strong at *K* ≈ 45 for both LP models.

### 3.5 FLOCK

**Figure 5:**
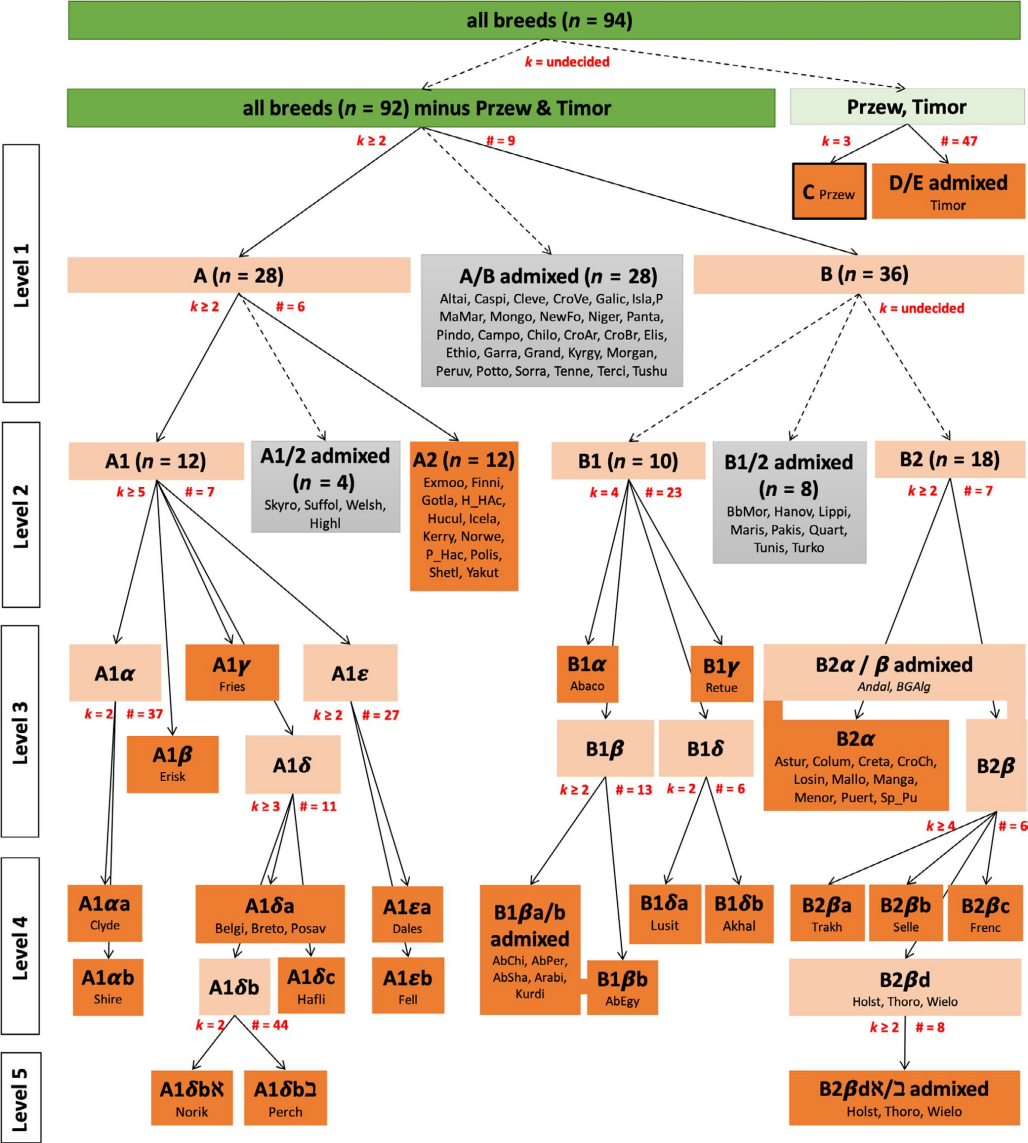
Summary of the FLOCK hierarchical analysis. Analyses were performed until there was no evidence for genetic substructure. On the top‐level of the hierarchical analysis the breeds were partitioned by identifying outliers from the sample likelihood map (Fig S3, Supporting information; highlighted in green). For all subsequent hierarchical levels, outliers were not observed and not used for decision‐making. Light red marks groups of breeds with genetic substructure and dark red marks non‐dividable groups without genetic substructure. In each case, when FLOCK produced an “undecided stopping criterion” when all breeds from a group were included but reached a definite stopping criterion after admixed breeds were excluded, these admixed breeds (highlighted with grey and a dashed arrow to the parental breed group) were excluded from subsequent analysis. Partitioning of groups due to reached a definite stopping criterion are indicated by unbroken arrows. The maximum plateau length count, #, and the optimal number of clusters, k, interpreted from the stopping conditions, are shown in red. In the case where admixed breeds were excluded, the # and k values, refer to the repeated FLOCK analysis after the exclusion. Groups of breeds are named according the hierarchical level of the analysis

No stopping condition was reached when all breeds were evaluated (Figure S5). The inspection of the *LLOD_K=2_* scores indicates the Przewalski horse and the Timor Pony as visually clear outliers. When analyzed separately, each showed a particularly long, and thus well supported, plateau length over 50 runs at *K = *3. The individuals were completely separated into three clusters with Przewalski occurring only in cluster C and Timor Ponies in clusters D or E (Figure 5).

After excluding both the Timor and Przewalski, the analysis of the 92 remaining breeds failed to produce a stopping condition again. The inspection of the sample allocation matrix separated 28 admixed breeds. Repeated analysis with the remaining breeds produces a stopping condition with cluster A, representing mainly ponies and the “Cold‐blood” group, and cluster B, representing mainly the “Warm‐blood” group. In total, six hierarchical levels were identified. Three groups of breeds with admixed ancestry were restricted to the top two levels and included with 40 breeds a substantial proportion (43%) of all investigated breeds. The 54 assigned breeds partitioned into 26 final clusters without any further detected substructure. Partitioning always occurred between breeds except for cluster B2βd. The three largest of the final clusters include a cluster of mainly ponies ranging from the British Isles to the Carpathian Mountains, A1, a cluster of horses mainly of Iberian origin, B2α, and an admixed cluster, B1βa/b, of Arab horses. The successful clustering of 54 breeds highlights that the 40 breeds that resulted in an “undecided stopping condition” are caused not by a global information deficiency of the used microsatellites but by admixture. When adding the admixed breeds one by one to a reanalysis, successful stopping conditions were reached in all cases but three. The total 26 clusters, that could not be further subdivided, correspond to the maximum optimal *K*, *K*_opt[FLOCK] _= 26. FLOCK’s separation of admixed and non‐admixed breeds at the hierarchical levels 1 and 2, in many cases, did not correspond with the cluster membership coefficients seen for STRUCTURE (Figure 5). This again shows the difficulty of working with a single species differentiated as breeds. Historically, many of these groups were crossed either accidentally or intentionally when horses from different regions were brought into contact by man. As well, many modern breeds, although considered distinct today, were created by crossing two or more breeds.

### 3.6 DAPC

**Figure 6:**
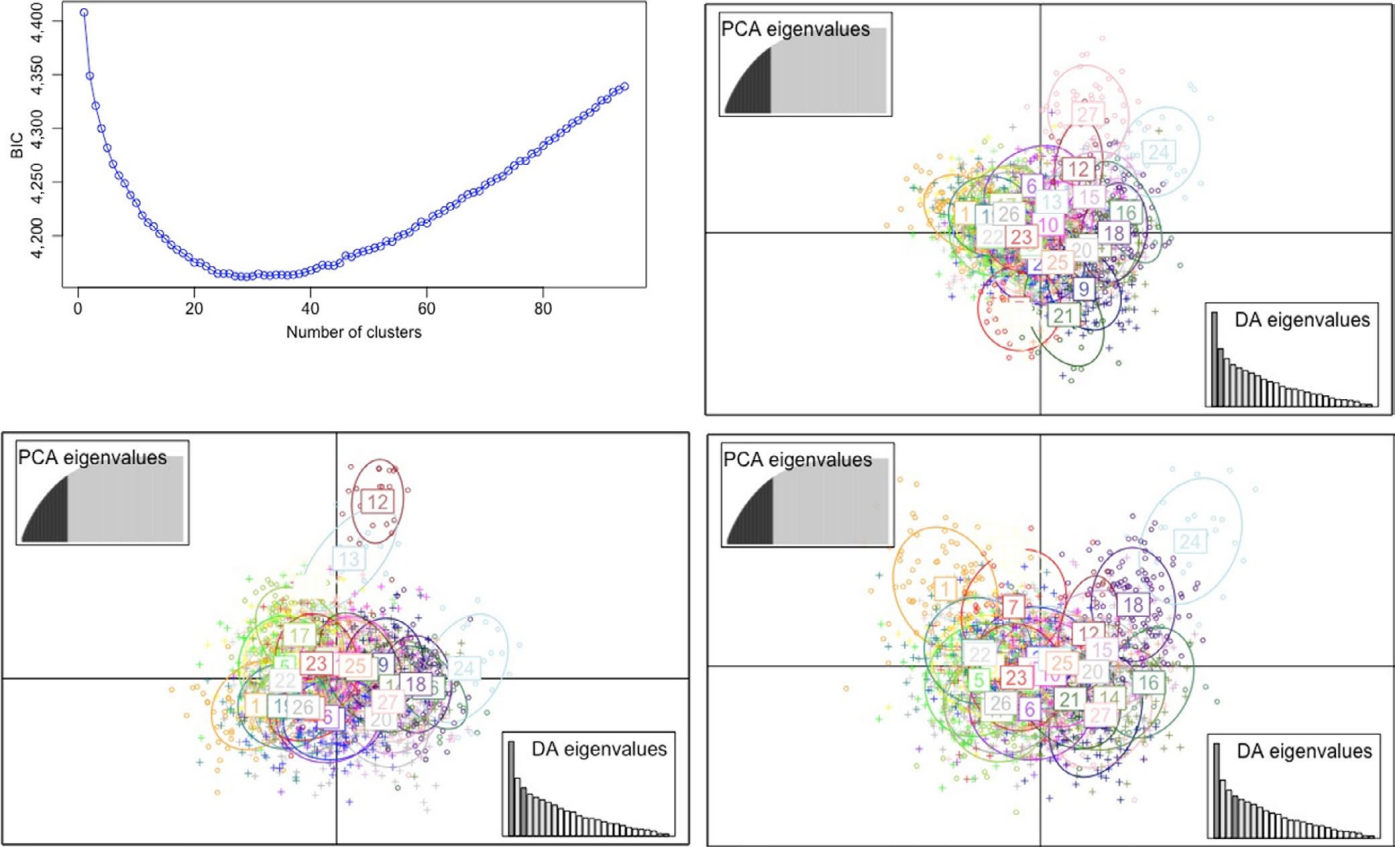
DAPC analysis with *K*=27 selected by Ward’s clustering method as the as the most parsimonious model describing the data set of 94 horse breeds. (A) Bayesian Information Criterion, BIC, values for all 94 clusters evaluated with BIC_*K*=27_ = 4162.3 indicated by an arrow. Scatter plots show the DAPC components 1 versus 2 (B), 1 versus 3 (C) and 1 versus 4 (D). Cluster membership of each individuals is depicted by distant colours inside their 95% inertia ellipses. Different symbols represent proportions of successful DA reassignment of individuals to their PCA clusters (circles for Prop_cluster_ > 0.75 and crosses for Prop_cluster_ < 0.75). The accumulated amount of variance explained by stepwise increasing number PA is shown in the inserted graphs at the top left of the scatter plots; the cut‐off point of the 50 PA used for the analysis represents 77.3 % of the total variance (shown in dark grey). DA eigenvalues are shown in the inserted graphs at the bottom right, whereby the numbers of the discriminants plotted against each other are indicated by dark grey and the eight discriminants retained for the analysis in light grey

The Bayesian information criterion (BIC) was minimal between approximately *K = *20 and *K* = 40 at the “elbow” of BIC values (Figure 6). While this range of *K* provides a useful parameter space to adequately describe the data, it did not provide a single, clear minimum BIC value at any *K*. Ward's clustering method differentiated *K = *27 as the model with the sharpest decrease of BIC values, indicating *K*_opt[DAPC]_ *__*=*_ _*27. Retaining the first 50 principal components and eight discriminants, eleven of the 27 *K*_opt[DAPC] _clusters successfully reassigned individuals by DA to their PCA clusters in over 75% of cases. This is mirrored in the DA scatter plots where some genotypic clusters separate clearly along the first four DA axes (Figure 6). The remaining 15 of the 27 *K*_opt[DAPC] _clusters had lower proportions of successful DA reassignment (0.34–0.74) and did not separate in the plots. Analyses retaining 75 PCs with 8 and 15 discriminants gave very similar results (not shown). The distribution of the *K* = 27 posterior cluster memberships in breeds is visualized in Figure 4, alongside the structure results. The visual pattern corresponds well with structure’s membership coefficients at *K* ≈ 13 for both LP scenarios. However, the posterior cluster memberships are strongly fragmented and reveal only strong genetic overlap between breeds that are known to be similar according to breed history (the Arabian horses, the Belgian/ Breton/ Haflinger group, and Mangalarga/Mangalarga Marchador, Figure 4b). However, groups of genetically associated breeds, especially the Argentinian, Brazilian, and Chilean Criollo breeds, are not identified as a breed group, which is in contrast to the results of PCoA and STRUCTURE at both LP scenarios, and the known breed histories.

## 4 DISCUSSION

Today, there are about 600 populations of horses that could be recognized as breeds (Hendricks, 2007). Analyses of nuclear genetic variation of 36 breeds using SNP markers have shown that most fall into five major breed groups (Petersen et al., 2013) and our results correspond with these categories. These are (a) Oriental breeds of with the true Arabian breeds as one subgroup and other Asian and Middle Eastern breeds as a separate subgroup; (b) European breeds east of Spain which show strong influence from the English Thoroughbred; (c) Iberian breeds from Spain and Portugal plus American breeds with strong Iberian backgrounds; (d) “cold‐blooded” horses of heavy draft breeds and true pony breeds which represent subgroups within this group; and (e) North American breeds which largely represent English and Spanish heritage with components of French, Dutch, and other European ancestry. Within these groups are breeds which do not well fit their group of origin due to severe loss of genetic diversity. Populations such as Abaco and the Sorraia in our study are good examples. However, fine‐scale population genetic structure remains unclear.

We used a large set of 94 breeds, covering an enormous amount of differing breed histories, to evaluate the recovery of such fine‐scale structure. The analysis of genetic population structure ultimately aims to recover the true, but unknown, underpinning processes leading to observed genetic patterns and, thus, the true but unknown number of population clusters. Besides understanding evolutionary processes, the evaluation is of concern for applied management as the resources available for conservation of endangered domestic breeds are quite limited. Thus, knowledge that two separate but similar populations are indeed different is critical for proper resource use. However, this is inherently difficult (Evanno et al., 2005; Pritchard et al., 2010). Our large empiric data set produced reliable mean *P(K)* estimates even with relatively low numbers of MCMC iterations and replicates. However, despite that our number of MCMCs (up to three million) and replicates (up to 100) is among the highest in the literature the ∆*K* estimator failed to converge, powerfully demonstrating the problems the ∆*K* statistics may face. How often this problem might have occurred in published studies remains unknown as studies have, in general, not evaluated the issue. The large observed variance between MCMC iterations within each evaluated *K* for both locprior models is the proximate reason for the poor performance of the ∆*K* method. Variance over *K* was as expected with mostly low values up to the plateau of the *P(K*) (Pritchard et al., 2000). We partitioned the replicated MCMC iterations in replicate blocks to evaluate the variance introduced by the MCMC characteristics. The variance between these replicate blocks was large and correlations between them were low. Consequently, the ratios between the highest ∆*K* peaks, that is, *K*_opt[Evanno]_, and the second highest peaks changed constantly over accumulated replicate blocks leading often to changes in *K*_opt[Evanno] _even at the tail end of the up to 100 conducted replicates. Especially worrisome is the observation that *K*_opt[Evanno] _appeared to have converged over long stretches of replicates in some cases, but then changed again, indicating a random walk characteristics of the MCMC procedure. In another case, *K*_opt[Evanno] _was stable over 40 replicates, but a recalculation of randomized blocks revealed different *K*_opt[Evanno]_ estimates. Thus, even apparently stable ∆K estimates might be misleading when variance and ratios between the highest ∆*K* peaks, that is, *K_opt[Evanno]_* and the second highest peaks are high. Possible factors underpinning the failure to converge on stable ∆*K* estimates include the general statistical aspects of the estimation procedure per se and how it is being applied, the biological aspects of population history and structure, and the sampling procedure.

Unequal sample sizes between populations, which can skew analyses (Puechmaille, 2016), are unlikely to have contributed to the observed problems of unstable *K*_opt[Evanno]_ estimates as we standardized sample sizes across all breeds. Furthermore, the analysis of the statistical power of the marker set across all test scenarios, which included the range of observed *F*_ST_ values, confirmed high power, making it unlikely that the marker set was insufficiently large to detect genetic structuring. Failure to reliably identify the main genetic clusters has been attributed to structure’s inherent feature of forcing genetic components into too few clusters (Kalinowski, 2011; Wang et al., 2007). A CLUMPAK pattern of multimodality indicates insufficient numbers of available clusters for the MCMC searching space to consistently assign genotypes to the same clusters and thus reaching unambiguous results (Wang et al., 2007). The random walk characteristic of MCMC processes may cause convergence at suboptimal solutions in the clustering space of possible genetic membership coefficients. However, the re‐application of the ∆*K* method for major modes still resulted in instable *K*_opt[Evanno]_ indicating that the presence of suboptimal solutions was unlikely the cause of the observed instability in the empiric data set. structure allocates some genetic subsets to spurious ghost clusters instead of leaving them empty, thus increasing the variance of the *P(K)* likelihoods.

The 15 autosomal microsatellite markers we applied have been in widespread use since they were recommended for diversity studies by ISAG‐FAO and the International Society for Animal Genetics (Cothran & Luís, 2005; FAO, 2011). Despite the advance of SNP genotyping, very few studies of horses have used SNPs to date for phylogenetic questions (Petersen et al., 2013) and the use of use of 12 to 17 microsatellites for breed analysis remains popular (e.g., Cosenza, La Rosa, Rosati, & Chiofalo, 2019; Isakova et al., 2019; Khanshour, Hempsey, Juras, & Cothran, 2019; Khaudov et al., 2019; Ustyantseva, Khrabrova, Abramova, & Ryabova, 2019; Zeng et al., 2019). The dendrogram of breed relationships analyzed by SNP typing of 36 breeds (Petersen et al., 2013) is almost an exact match of the results from the 15 microsatellite loci, thus suggesting that the results from our 15 microsatellites are robust. Increase of microsatellite numbers would likely allow higher resolution in the analysis of the genetic architecture of horses as has been observed in numerous other cases (e.g., in chicken, Gärke et al., 2012). Whether a higher number of loci would increase or decrease the performance of the MCMC searches of the parameter space for large sample sets needs to be evaluated.

### 4.1 Biological aspects of population history and structure

The evolutionary history and genetic structure of horses might represent a particularly difficult case for structure to solve. The relationships among domestic breeds are complex due to a high degree of mixing over generations. Horses were domesticated largely for their transportation abilities, and this ability has been widely used for at least the past 4,000 years. As horses were moved from one place to another, there was interbreeding of the invading horses with the resident horses. Widespread bidirectional gene flow and reticulate events persisted during and after domestication including the Przewalski and an extinct wild, taxonomically undescribed horse population (Der Sarkissian et al., 2015; Pardi & Scornavacca, 2015; Schubert et al., 2014; Warmuth et al., 2012). A strong sex bias during domestication led to differential maternal and paternal contributions to the overall gene pool and characterizes the founding and improvements of modern breeds especially since the formation of the earliest studbook (Lipizzaner in 1580, Galov et al., 2013; Wallner et al., 2017). The complex breed relations are also exemplified by the co‐existence of closed breeds without admixture from outside breeds, open breeds with admixture, and different natural and artificial selective pressures. Variation in mtDNA control region sequences gives a clear example of this crossbreeding. Phylogenetic trees typically resulted in low bootstrap values and thus low statistical support irrespective of tree‐building and distance algorithms (Conant et al., 2012; Cothran & Luís, 2005; Pires et al., 2016). An epiphenomenon of the highly complex genetic basis appears to be the lack of a clear separation of breeds at low *K* and the lack of breed separation of breed membership coefficients at very high *K*. In contrast, such strong clustering was observed in domestic sheep, which are by far less intensively managed than horses (Grégoire Leroy et al., 2015). Extensive human data sets representing the complex migration and dispersal patterns during and after the peopling of the Americas have also demonstrated that it is difficult to adequately infer genetic structure for complex data (Corander et al., 2008; Wang et al., 2007).

Although structure reveals which breeds are largely admixed, it does not reveal which breeds contribute to the large variances of *P(K*). In contrast, the flock results pointed at the admixed breeds which prevented the algorithm to find solutions for genetic structuring. Excluding them, breed clustering could be identified on a hierarchical level. The flock algorithm has previously been criticized, especially for large data sets, because the reliance on inference rules regarding its “plateau record” is regarded as “not helpful” (Anderson & Barry, 2015). Indeed, the rules appear arbitrary, but the main strength is the important evaluation of the consequences of low information content of the marker system to find reliable solutions (Duchesne & Turgeon, 2016; Duchesne & Turgeon, 2012; Orozco‐terWengel, Corander, & Schloetterer, 2011; Putman & Carbone, 2014). In contrast, the quantitative decision‐making process of the ∆*K* method appears statistically elegant, but it always produces a “solution” whether or not the solution is adequate. The additional, so far not utilized, strength is flock's ability to identify whether failure to identify population structure is based on high levels of admixture.

### 4.2 Sample structure

structure performs best with a small number of discrete populations (Pritchard et al., 2000), possibly reflecting that MCMC search algorithms face increasing difficulties to find stable solutions when searching complex and large parameter spaces. Although the analysis of a smaller subset of horses and breeds indeed demonstrated convergence at *K*_opt[Evanno]_ values, the number of samples appears not to be the crucial factor per se for consistently finding a solution in the large parameter spaces. This was demonstrated by our reanalysis of the large data set of Anacapa Island mice with eleven population samples and 1,361 individuals, where the increase of the originally published *R* = 20 repetitions to *R* = 100 did not produce any change in the *K*_opt[Evanno]_ estimate. Thus, the crucial factor appears to be the large number of populations and the genetic relationships between them. Large samples of populations can increase the possibility to include complex structures and reticulate events represented in the sample. This is especially true for domestic animals where large variation of breed origin, managed breed relationships, natural and artificial selection, selection of founder animals, population size, and demographic history is common.

Most studies of horses that have utilized STRUCTURE have examined a small number of breeds, often from a specific geographic locality (Barcaccia et al., 2013; Berber et al., 2014; Bömcke, Gengler, & Cothran, 2011; Conant et al., 2012; Cothran et al., 2011; Galov et al., 2013; Janova et al., 2013; Khanshour, Conant, et al., 2013; Khanshour et al., 2015; Koban et al., 2012; Kusza et al., 2013; Lopes et al., 2015; Mackowski, Mucha, Cholewinski, & Cieslak, 2015; Mujica, 2006; Pablo Gómez et al., 2017; Pires et al., 2016; Prystupa, Juras, Cothran, Buchanan, & Plante, 2012; Rendo, Iriondo, Manzano, & Estonba, 2012; Sereno, Sereno, Vega‐Pla, Kelly, & Bermejo, 2008; Tozaki et al., 2003; Uzans, Lucas, McLeod, & Frasier, 2015). The ∆*K* obtained in these studies appeared reasonable for the number of breeds. In some cases, a specific breed was compared to a large number of other breeds in a phylogenetic analysis but the breeds used in their STRUCTURE analysis were a subset of those in the phylogenetic analysis based upon what was found with the phylogenetic tree (Khanshour et al., 2015). In other cases, a small number of closely related breeds were analyzed to reveal fine structure within the group which could indicate differences in the histories of the individual breeds within the group (Khanshour, Conant, et al., 2013; Khanshour, Conant, et al., 2013).

### 4.3 Recommendations

First, the possibility of interpretation based on false ∆*K* estimates is real because ∆*K* will always give a solution even with a very small number of replicates. Convergence should be evaluated by running many more replicates than the currently recommended *R* = 20. Care must be taken that the random walk characteristics of the MCMC algorithm can falsely suggest convergence even with larger numbers of replicates. The inspection of the ratios between the highest and second highest ∆*K* peaks will indicate the overall variance in the estimates and guide the choice of *R*.

Second, the evaluation of convergence of ∆*K* using replicate blocks is a suitable technique to choose and to justify the final number of replicates. So far, the approach taken in the literature to check for convergence of STRUCTURE parameters focusses on single MCMC iterations and a visual inspection of key parameters, in particular the posterior probability of the data for a given *K* (Gilbert et al., 2012; Pritchard et al., 2010). However, this inspection is qualitative as there is no definition of when convergence has been achieved and how much variance is acceptable. In the literature, it is rarely applied or reported. In contrast, the monitoring of ∆*K* over repeats is a more stringent and defined approach, whether ∆*K* is subsequently being used for decision‐making or not.

Third, we suggest to identify a range of *K* that might feasibly explain the data well instead of using point estimates such as ∆*K*, corrected ∆*K* estimators (Puechmaille, 2016), or the Pritchard estimator. The over‐reliance on *K*_opt_ and the enforcement of a specific value *K*_opt_ irrespective whether Δ*K* has converged can be inadequate and should be replaced by a qualitative description of clustering over increasing *K*, which appears scientifically more honest. This adds strength to the increasing number of studies which forfeit the estimation of an optimal *K* and describe STRUCTURE results over *K* in relation to known natural history data from populations or breeds (e.g., Cortés et al., 2017 in horses; Leroy et al., 2015 in sheep). Although these studies do elaborate why point estimators have not been used, the underpinning rationale is to exercise precautionary care. Because the ∆*K* method identifies the uppermost level of clustering only, an over‐reliance on this method might cause the missing of more subtle patterns. Again, this is an indication that subsets of the total breed set may offer a superior method of analysis. When cluster numbers above the maximum ∆*K* value are being interpreted from the membership plots, then ∆*K* appears to be of relatively little importance. A combination of those *K* values that neither produce CLUMPAK minor modes nor ghost clusters points to well‐fitting MCMC solutions which neither enforce a too small number of clusters (minor modes) nor too many clusters (ghosts). They can then guide the subsequent interpretation in the context of known information on the populations or breeds. This approach has the major advantage that the overall variance between MCMC estimates is relatively unimportant because these parameters rely on mean *P(K)* only. The estimates of the means are very stable even at small numbers of repeats and MCMC iterations. Mean *P(K)* values for the horses converged relatively fast for all MCMC/‐LP scenario, for example, a correlation of >0.98 compared between 10 and 15 replicates and compared to the other MCMC scenarios. Thus, the computing time can be dramatically reduced from the normally prohibitive extensive times for large data sets. A faster structure version is currently available only for SNP data and not microsatellites (Raj, Stephens, & Pritchard, 2014).

Fourth, we recommend to check whether the large‐scale analysis clusters populations into smaller units that are consistent with known population processes such as breed histories. If this is the case, we suggest that these small‐scale clusters are then analyzed separately. As in our case of Arabian breeds, the analysis of small numbers of sample populations is faster and performs better than the larger sample sets (Pritchard et al., 2000).

Finally, when the data or the known natural history indicate complex population relationships, it is advisable to augment Bayesian analysis with dapc and flock in order to check for consistencies and to better evaluate the data from different methodological angles. However, it must be noted that dapc clearly did not characterize population subdivision better than structure as evidenced by the failure to recover the similarities of the South American Criollo breeds, where structure’s results corresponded well with the known historic breed origin. Although dapc revealed similar qualitative results as pcoa and revealed the same major breed clusters as structure, it did not add further information as provided by the other methods. The usefulness of flock for assessing the power of the concretely used genetic markers set has been pointed out previously (Putman & Carbone, 2014) but it is rarely applied (only 30 citations in web of science up to June 2017). Its additional major advantage is that it allows to assess whether failure to successfully cluster populations is caused by the breed relationships themselves. We describe the corresponding strategy here (Duchesne, pers. communication) as it was not described in the publication describing FLOCK or the current manual (Duchesne et al., 2013; Duchesne & Turgeon, 2012).

## CONFLICT OF INTEREST

None declared.

## AUTHOR CONTRIBUTIONS

EGC collected the data and provided ideas during analysis and writing of the manuscript. SMF performed the analyses and wrote the manuscript. SG, RJ, AK, FL, CL, AMM, AMM, FM, MMO, LO, YMS and JLVP provided samples. All authors provided ideas and contributed to the manuscript writing.

## Supporting information

Table S1

Table S2

Figure S1

Figures S2

Figure S3

Figure S4

Figure S5

## Data Availability

Microsatellite genotypes are available from Dryad (https://doi.org/10.5061/dryad.tmpg4f4vh).
