# Supplementary figures and images for: Major inconsistencies of inferred population genetic structure estimated in a large set of domestic horse breeds using microsatellites
Source: Ecol Evol. 2020 Apr 12;10(10):4261–79. doi: 10.1002/ece3.6195 (PMC7246218; doi:10.1002/ece3.6195)

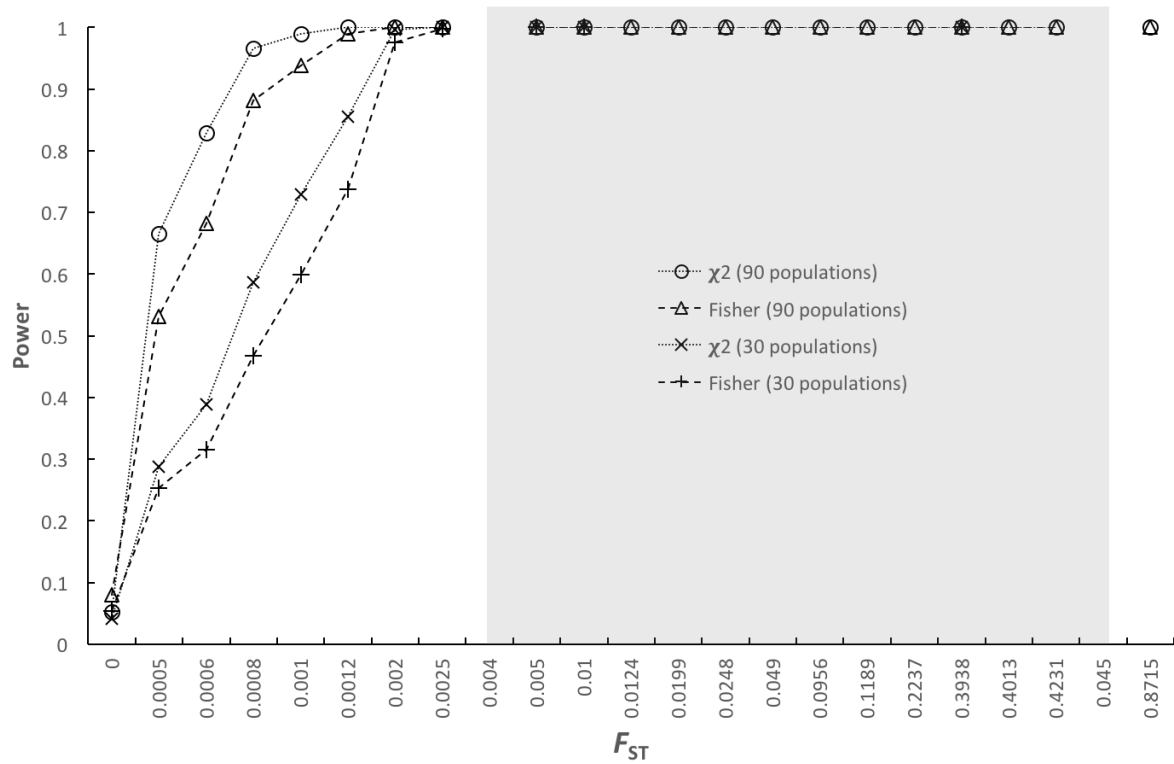

Supplement: Supplementary file 3 — Figure S1 [file ECE3-10-4261-s003.pdf]

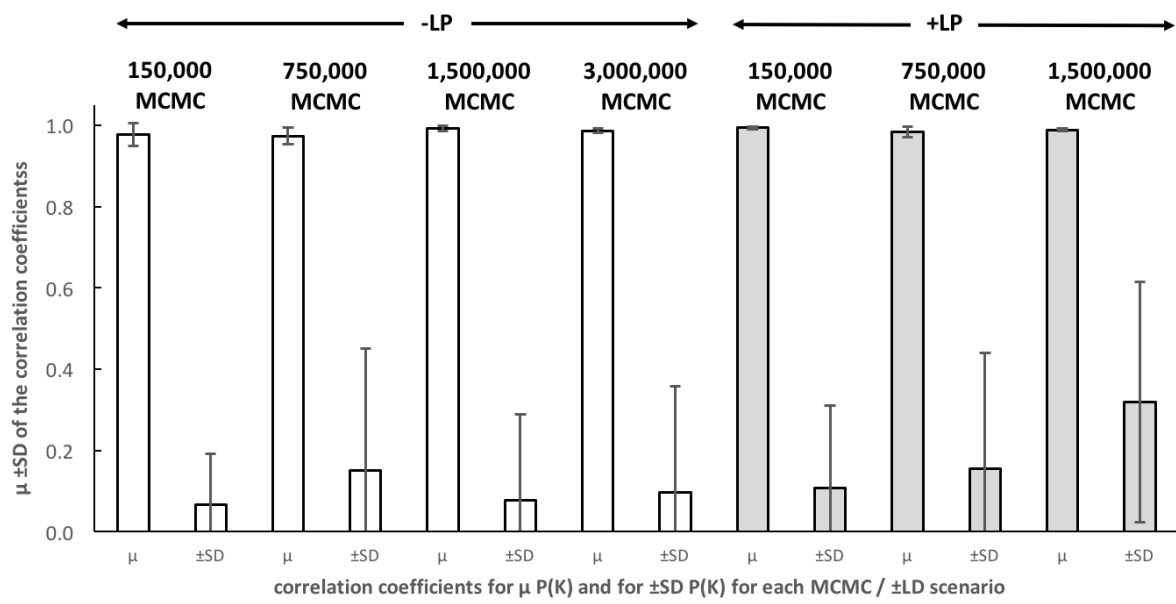

Supplement: Supplementary file 4 — Figures S2 [file ECE3-10-4261-s004.pdf]

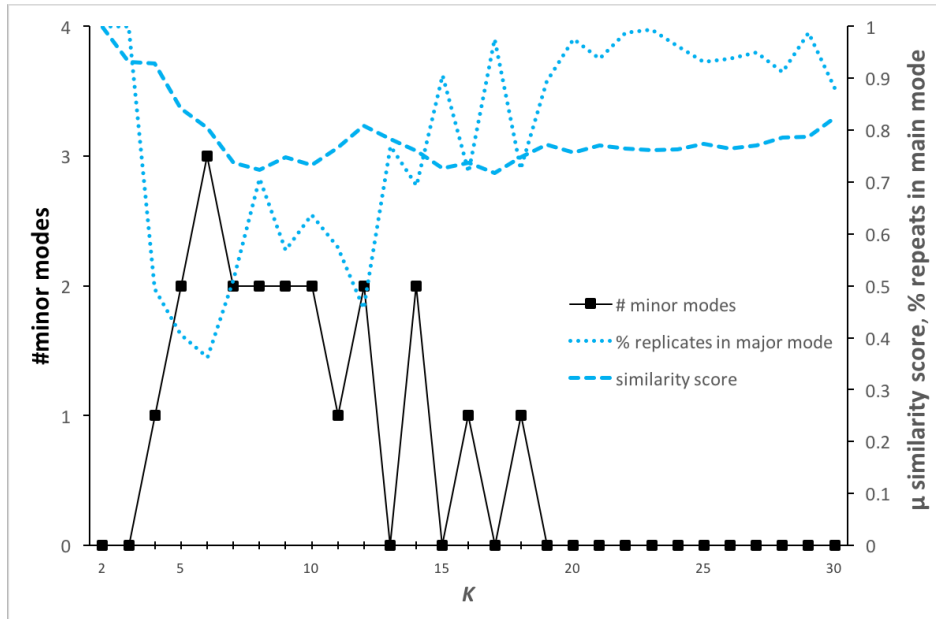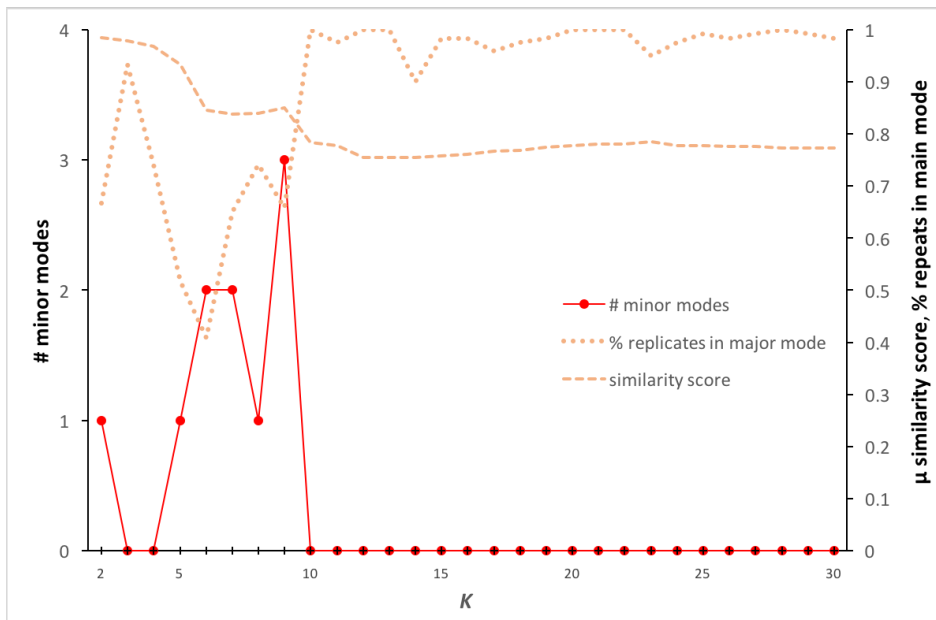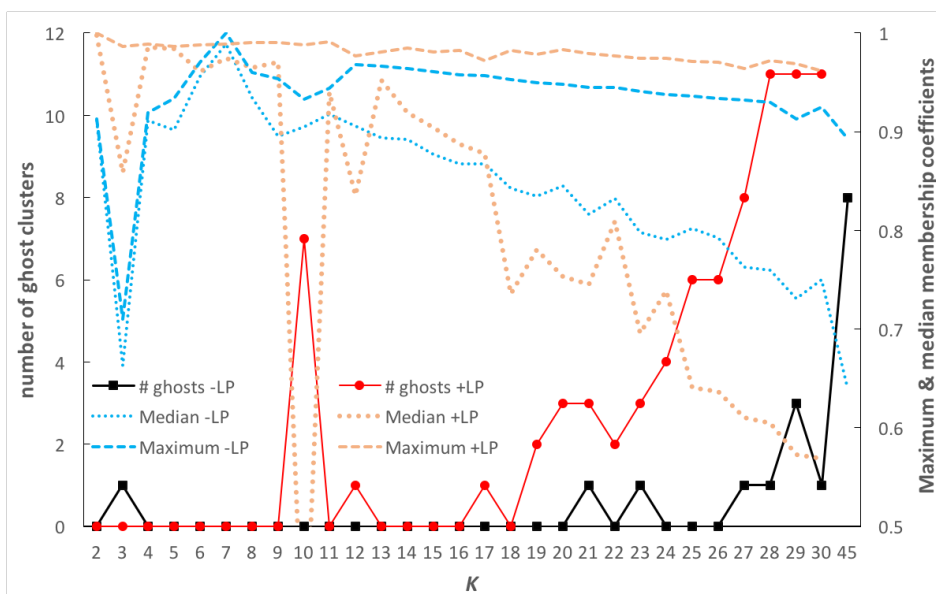

Supplement: Supplementary file 5 — Figure S3 [file ECE3-10-4261-s005.pdf]

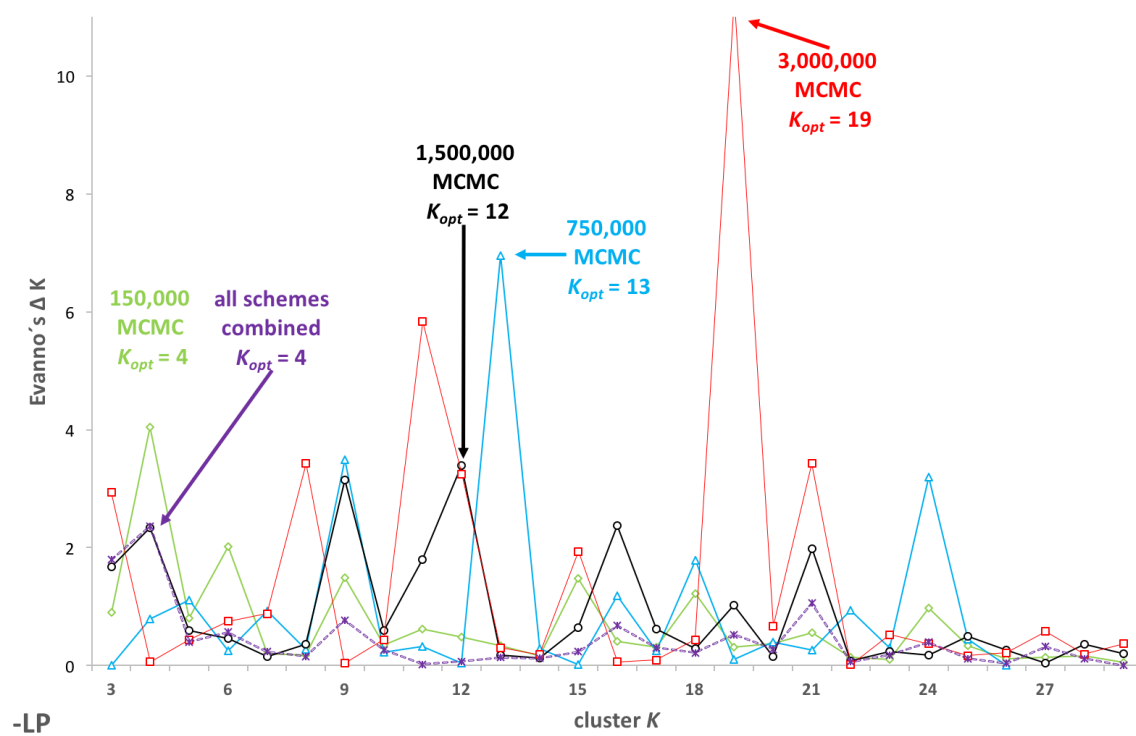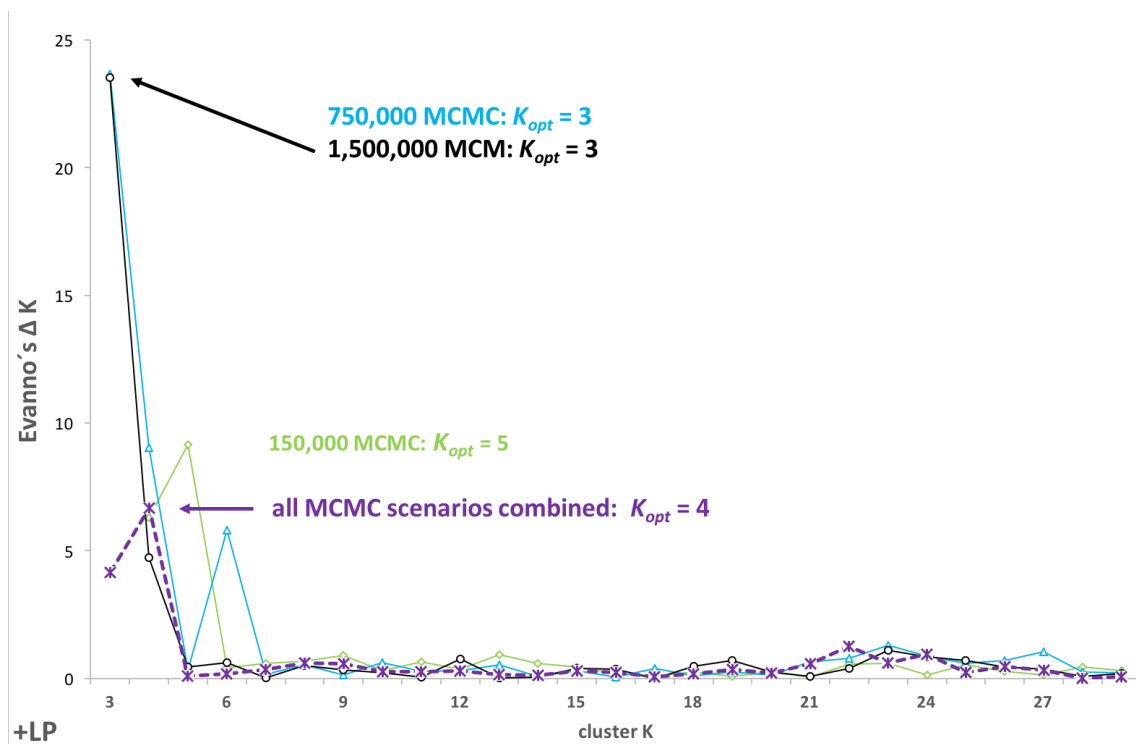

Supplement: Supplementary file 6 — Figure S4 [file ECE3-10-4261-s006.pdf]

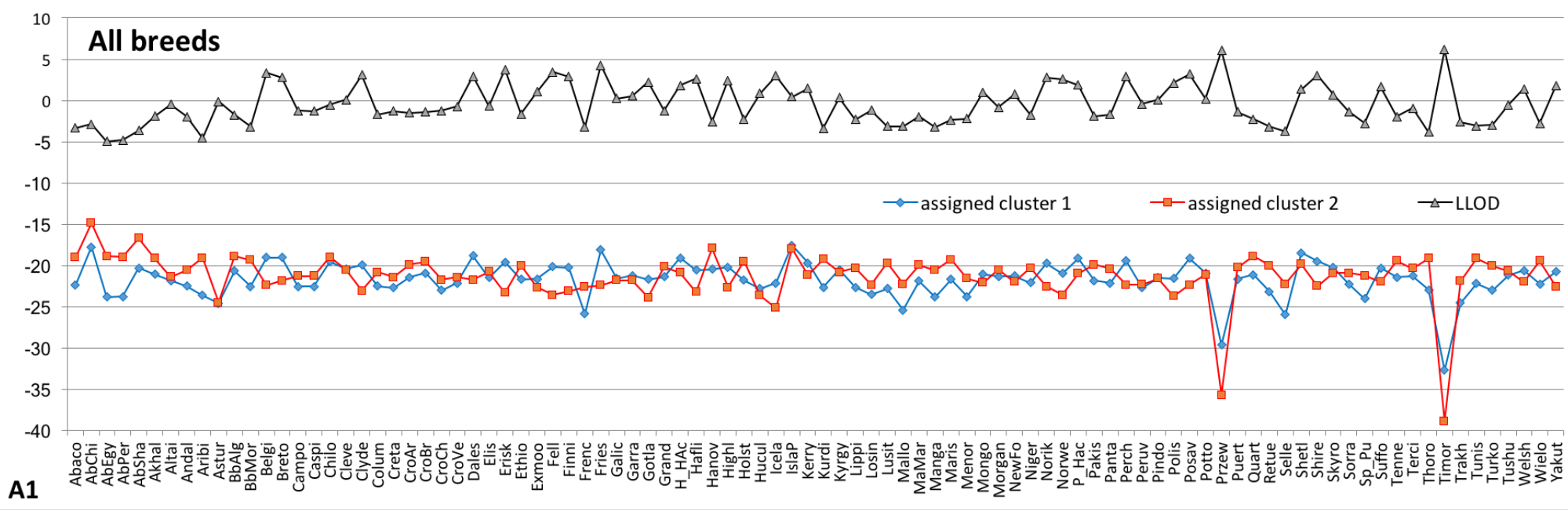

A1

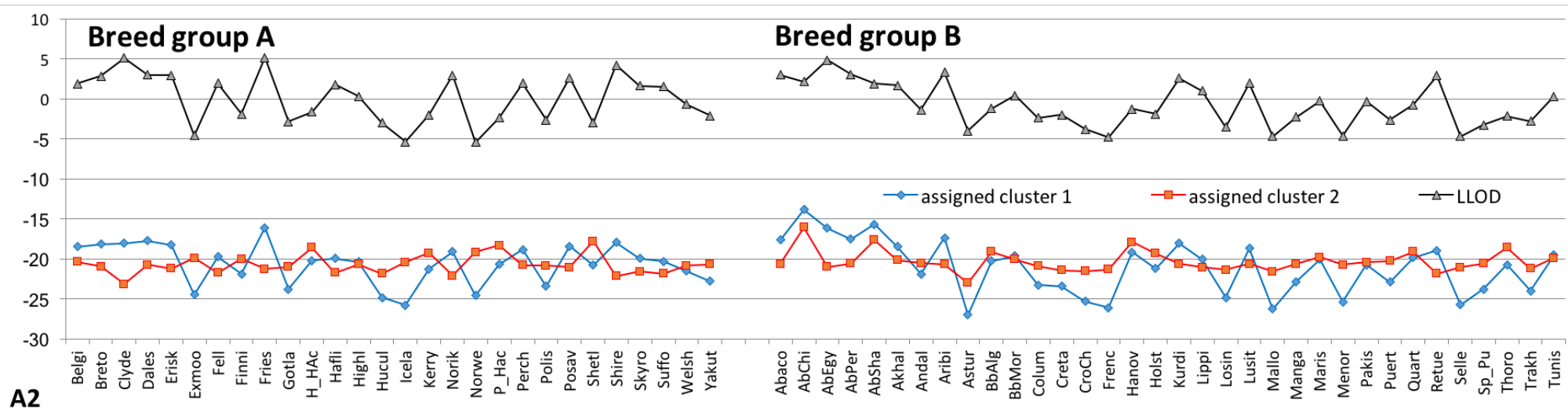

A2

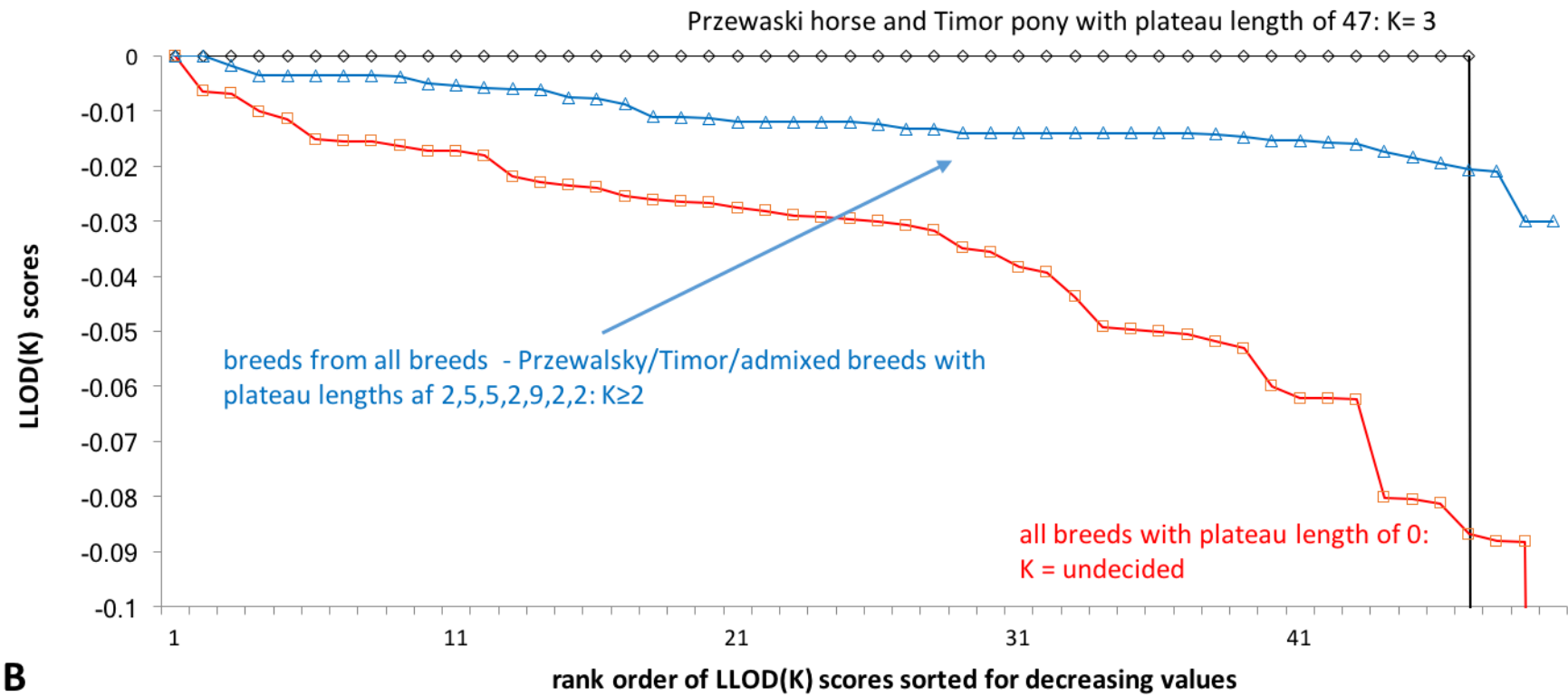

B

Supplement: Supplementary file 7 — Figure S5 [file ECE3-10-4261-s007.pdf]
